# Quantitative and Rapid DNA Detection by Laser Transmission Spectroscopy

**DOI:** 10.1371/journal.pone.0029224

**Published:** 2011-12-16

**Authors:** Frank Li, Andrew R. Mahon, Matthew A. Barnes, Jeffery Feder, David M. Lodge, Ching-Ting Hwang, Robert Schafer, Steven T. Ruggiero, Carol E. Tanner

**Affiliations:** 1 Department of Physics, University of Notre Dame, Notre Dame, Indiana, United States of America; 2 Department of Biological Sciences and Center for Aquatic Conservation, University of Notre Dame, Notre Dame, Indiana, United States of America; 3 Department of Biology and Institute for Great Lakes Research, Central Michigan University, Mount Pleasant, Michigan, United States of America; Northeastern University, United States of America

## Abstract

Laser transmission spectroscopy (LTS) is a quantitative and rapid in vitro technique for measuring the size, shape, and number of nanoparticles in suspension. Here we report on the application of LTS as a novel detection method for species-specific DNA where the presence of one invasive species was differentiated from a closely related invasive sister species. The method employs carboxylated polystyrene nanoparticles functionalized with short DNA fragments that are complimentary to a specific target DNA sequence. In solution, the DNA strands containing targets bind to the tags resulting in a sizable increase in the nanoparticle diameter, which is rapidly and quantitatively measured using LTS. DNA strands that do not contain the target sequence do not bind and produce no size change of the carboxylated beads. The results show that LTS has the potential to become a quantitative and rapid DNA detection method suitable for many real-world applications.

## Introduction

Laser transmission spectroscopy (LTS) [1] is a new technique capable of rapidly determining the size, shape, and number of nanoparticles in suspension. Here, we present new results demonstrating a novel application of LTS technology as a DNA diagnostic tool. Successful DNA detection can impact many important endeavors such as invasive-species research, medical diagnostics, drug development, environmental health, and the search for exotic life forms. The ability to rapidly and quantitatively distinguish between target and non-target organisms at the point of contact is a critical challenge for many DNA detection protocols. For example, invasive species cost the US hundreds of billions of dollars annually in agriculture losses, environmental harm, and disease outbreaks [2], [3]. Invasions could potentially be prevented and/or managed more efficiently if detected early in the field. DNA detection also represents an important tool in understanding and indicating the presence of genetic diseases such as cancer [4].

Established techniques for DNA detection and genic profiling fall into a few broad categories. These include gel electrophoresis, fluorescence approaches, and lab-on-chip methods. The lab-on-a-chip methods include various combinations of nanochannels, microfluidics, and microarrays along with observations made by electronic, visual, or fluorometric means. With fluorescence approaches the amount of DNA in the sample can range from 3.8×10^13^ to 1.5×10^17^ nucleotides/mL, while the other methods typically require >10^17^ nucleotides/mL. Due to the quantity of DNA required, these techniques often still depend on polymerase chain reaction (PCR) as a first step. In general, these techniques have limitations due to high cost, relatively low throughput in terms of sample number and detection time, and high dependence upon sample preparation. Related to DNA detection is the question of whether PCR amplification as a required first step can be eliminated. Work in this area by members of this team and others has included systems based on carbon nanotubes [5],[6],[7], microfluidic streams [8], [9], silicon nanowire sensors [10], nanoparticle multilayers [11], magnetic nanobeads [12], organic transistors [13], motion-based sensors using catalytic nanowires [14], functionalized hydrogels or nanoparticles [15], DNA sandwich assays [16], and nanowire arrays [17]. Accordingly, there is much still to be gained from improvements in DNA detection technology. Whereas the portability, functionality, and reliability of these approaches in the field remain to be seen, based on our experience, laser transmission spectroscopy (LTS) represents a promising new approach for PCR elimination in the field setting.

## Materials and Methods

**Figure 1:**
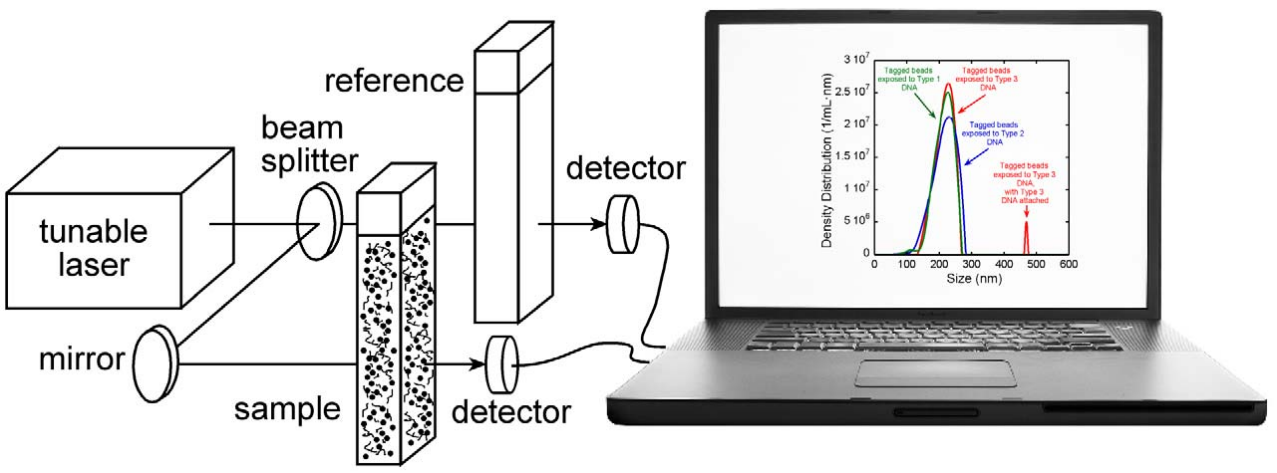
Schematic diagram of DNA detection using LTS.

**Figure 2:**
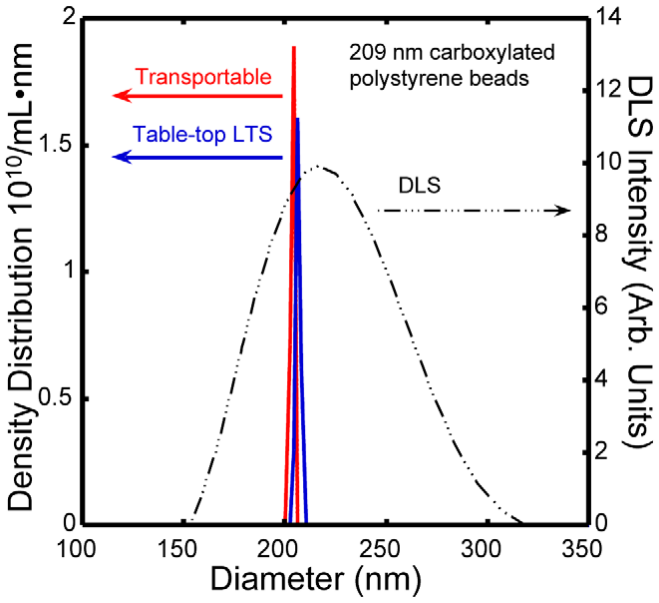
Comparison between LTS and DLS results. The plot shows the particle size distributions obtained for 209 nm carboxylated polystyrene beads in water using: the original table-top LTS apparatus (solid blue line); a transportable LTS based instrument (solid red line); and a commercial DLS based instrument (dash-dot-dot-dot line).

LTS is based on measuring wavelength-dependent light transmittance through a sample containing nanoparticles in suspension whereas other light based nanoparticle characterization techniques rely on diffraction and/or scattering [18], [19], [20]. A schematic diagram of our experimental approach is shown in Fig. 1, and Ref. 1 describes the apparatus and data analysis in detail. Briefly, the transmission of light through a sample cell containing particles plus suspension fluid is recorded along with that of a similar cell containing only the suspension fluid. The fundamental data-acquisition process involves measuring the wavelength-dependent transmission of light (quantified as extinction) through an aqueous suspension of nanoparticles. Here, the pertinent wavelength range is from ∼300 to 1000 nm. Given the extinction information, and the known wavelength-dependent properties of the beads, Mie theory can be used to accurately determine the bead diameter. The extinction data are analyzed and inverted by a computer algorithm that outputs the particle size distribution. Fig. 2 shows the LTS particle size distribution obtained for the 209 nm carboxylated polystyrene beads used in these measurements. The LTS measurements were done in two ways: first with our original LTS table-top apparatus having an acquisition and analysis time of ∼1 hour (solid blue line); and second with an automated transportable LTS based apparatus having a data acquisition time of ∼100 ms and analysis time of ∼1 min (solid red line). The LTS distributions are narrow (FWHMs of 2.9 nm table top, and 2.5 nm transportable) and quantitative (area under the curves of 5.1×10^10^ and 5.2×10^10^ particles/mL respectively, *i.e.* ∼0.5 nanomolar). Fig. 2 also shows the particle size distribution obtained using the common light scattering technique, dynamic light scattering (DLS) also known as photon correlation spectroscopy (PCS), employed in many commercially available particle size analyzers (FWHM 85 nm). As shown in Fig. 2, LTS has at about thirty times higher resolution and the capability of quantitatively determining the number density of nanoparticles in solution. In contrast, DLS has a much broader instrument function and can only produce a relative particle size distribution. The sensitivity limit of LTS reported in Ref. [1] for 1025 nm polystyrene spheres is ∼3580 particles/mL (*i.e.* 3.5×10^−17^ molar), which is 10^6^ times more sensitive than DLS for the same particles. The precision, accuracy, sensitivity, and resolution of LTS using NIST traceable polystyrene particles are detailed in Li *et al.* [1] where these properties are quantified for the size range important for DNA detection (∼50–1000 nm). The quantitative and rapid features of LTS may prove to be advantageous for many DNA detection applications [21] especially those requiring a transportable field compatible instrument such as invasive species detection.

The work presented here utilizes carboxylated polystyrene nanobeads functionalized with species-specific oligonucleotides (tags) that bind to species-specific DNA sequences (targets). LTS has more than sufficient resolution (3 nm for mixtures) to detect the large diameter increase (100 s of nm) that occurs when DNA strands containing targets hybridize with tags on the surface of the functionalized nanobeads. With LTS, the number of beads and their change in diameter are quantifiably measured. Two closely related invasive mussels were used in these studies to demonstrate the selectivity of LTS with respect to target and non-target DNA sequences. The data show that LTS can distinguish a species-specific DNA sequence of the invasive quagga mussel (*Dreissena bugensis*) from that of the evolutionarily related sister species, zebra mussel (*Dreissena polymorpha*), and the common planktonic cladoceran, (*Daphnia magna*). To demonstrate the general efficacy of LTS for DNA detection, the work presented here uses pre-screened PCR amplified mitochondrial DNA fragments from quagga mussels as targets.

**Figure 3:**
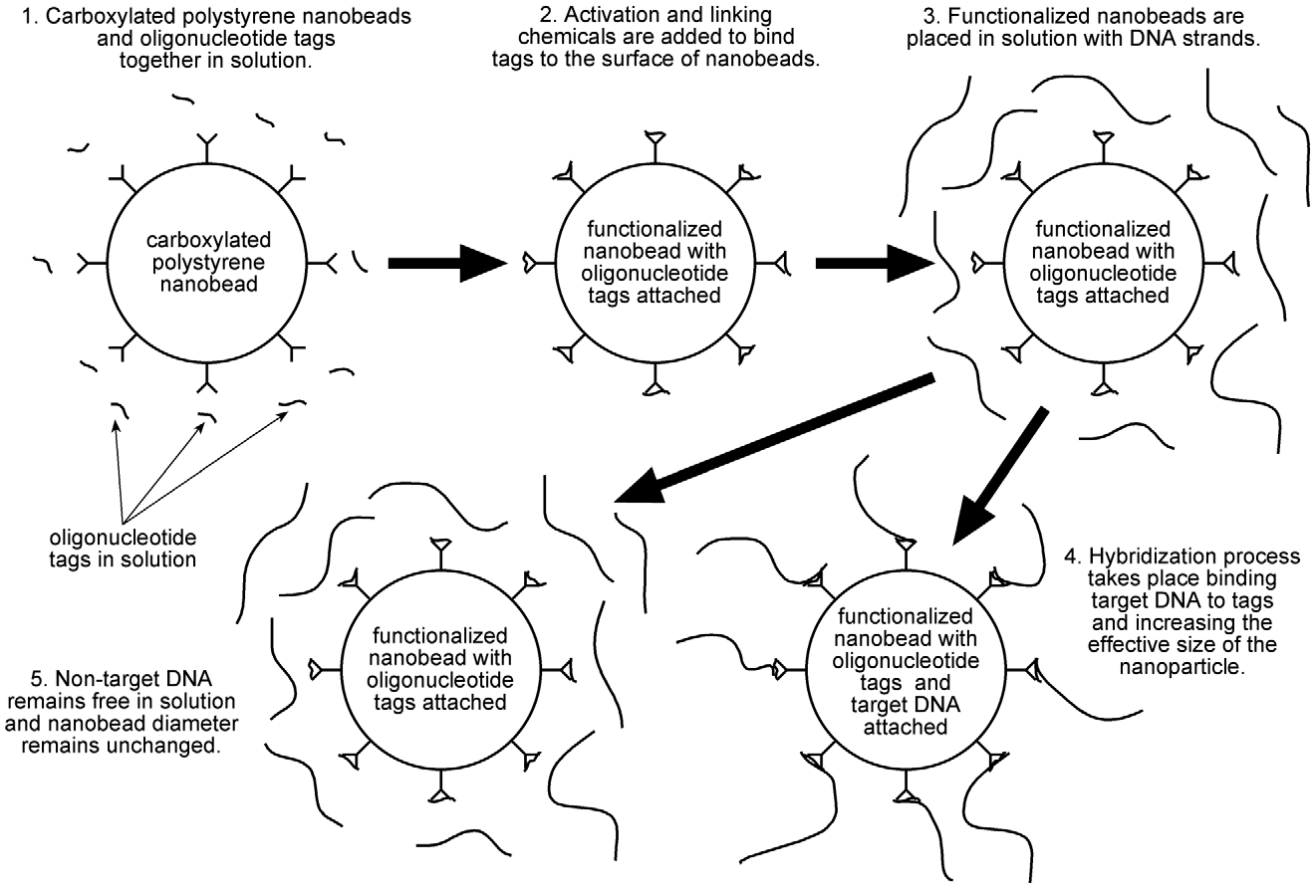
Schematic diagram of nanobead preparation (Steps 1 and 2) and binding of DNA to the functionalized beads (Steps 3–5).

Polystyrene was selected because of the availability of uniformly sized nanobeads of this material [22], [23]. Carboxylated polystyrene beads with a manufacturer's stated diameter of 209 nm were chosen because this size is well within LTS's operational range, and the expected diameter change would be significant and easily detected. The carboxyl groups on the surface of the beads were activated with 2-(N-morpholino) ethanesulfonic acid (MES) buffer at pH 6.0. A linker carbodiimide, 1-ethyl-3-(3-dimethylaminopropyl), was added to the bead solution to provide amino groups that covalently bond to both the carboxyl group of the beads and the carboxyl terminus of a species-specific tag. Constant agitation with the addition of ethanolamine was used to quench the conjugated beads after functionalization. See Fig. 3, steps 1 and 2. The prepared beads were stored in a buffer solution at 4°C to maintain separation and suspension prior to their use [24].

The tag used for functionalizing the beads is a 28 base oligonucleotide that is species-specific to the quagga mussel (*D. bugensis*). The biomarker is within the mitochondrial cytochrome c oxidase subunit I (COI) gene (See Table 1). Across the 28 bases of the tag, the quagga mussel (target species) differs by 7 nucleotides from the zebra mussel (*D. polymorpha* non-target species) and by 12 nucleotides from the common cladoceran (*Daphnia magna* also a non-target species). In Table 1, the differences between target and non-target sequences are bold and underlined. The biomarkers were previously published by Mahon *et al.* [25].

**Table 1:** Comparison between species-specific oligonucleotide tags and biomarkers where the differences are bold and underlined.

species description	DNA biomarker and 28-base sequences(A = adenine, C = cytosine, G = guanine, T = Thymine)
species-specific tag for quagga	A C A A G T T G G G G G T G G T T T A G G C G G G A G T
quagga mussel*(D. bugensis)* target	T G T T C A A C C C C C A C C A A A T C C G C C C T C A
zebra mussel*(D. polymorpha)*	**G** G T T C A A C C **A** C C **C** C C **G** A A T C C **T** C C **T** T C **C**
cladoceran*(Daphnia magna)*	**A** G T T C A A C C **A** **GTC** C C A **G** **CA** C C **A** C **T** **T** T C **C**

Genomic DNA used for Polymerase Chain Reaction (PCR) amplification was extracted from quagga mussel and zebra mussel muscle tissue and from the whole cladoceran organism using a Qiagen DNEasy extraction kit (Qiagen, Inc.). PCR amplification was performed on each extraction as described by Mahon *et al.* [13] using universal invertebrate primers [HCO-2198 and LCO-1490; xiv] (Table 2). In brief, PCR reactions consisted of 1 uL of genomic DNA, 0.75U Taq polymerase and 10X PCR buffer (5 Prime, Inc.), 2.5 mM Mg(OAc)2, 10 nmol of each dNTP, primers (final concentration 0.2 mM; Table 2), and deionized water for a total reaction volume of 25 uL. The PCR thermal program consisted of an initial denaturation step for 1 minute at 94°C followed by 30 cycles of 30 seconds at 94°C, 45 seconds at 48°C, and 1 minute at 72°C, then a final elongation for 8 minutes at 72°C. This reaction targeted and exponentially amplified a ∼600 base pair section of the mitochondrial cytochrome c oxidase subunit I gene for both target and non-target species.

**Table 2:** Molecular markers (primers) utilized for PCR amplification.

Species	Forward Primer	Reverse Primer
quagga mussel*(D. bugensis)*	(quagga COI-F)5′-CCTTATTATTCTGTTCGGCGTTTAG-3′	(HCO-2198)5′-TAAACTTCAGGGTGACCAAAAAATCA-3′
zebra mussel*(D. polymorpha)*	(LCO-1490)5′-GGTCAACAAATCATAAAGATATTG-3′	(HCO-2198)5′-TAAACTTCAGGGTGACCAAAAAATCA-3′
cladoceran*(Daphnia magna)*	(LCO-1490)5′-GGTCAACAAATCATAAAGATATTG-3′	(HCO-2198)5′-TAAACTTCAGGGTGACCAAAAAATCA-3′

After the PCR reactions were completed, the PCR products (double stranded DNA) from each organism was denatured by heating to 95°C for 2 minutes, then immediately chilled on ice for 2 minutes. Following this, 10 uL of each were combined with 20 uL of functionalized beads (concentration 1.04×10^9^/mL) at 48°C for one minute (Fig. 3, Steps 3 –5). The three samples containing DNA-plus-beads were placed in separate quartz spectrometer cells and analyzed by LTS with respect to a reference cell containing all the components used in preparing the DNA-plus-bead samples, excluding the DNA and the tagged beads. A control sample, which contained the tagged beads without DNA, was also run with respect to the same reference sample. In Li *et al.*, we discuss the details of LTS theory and operation [1].

## Results

**Figure 4:**
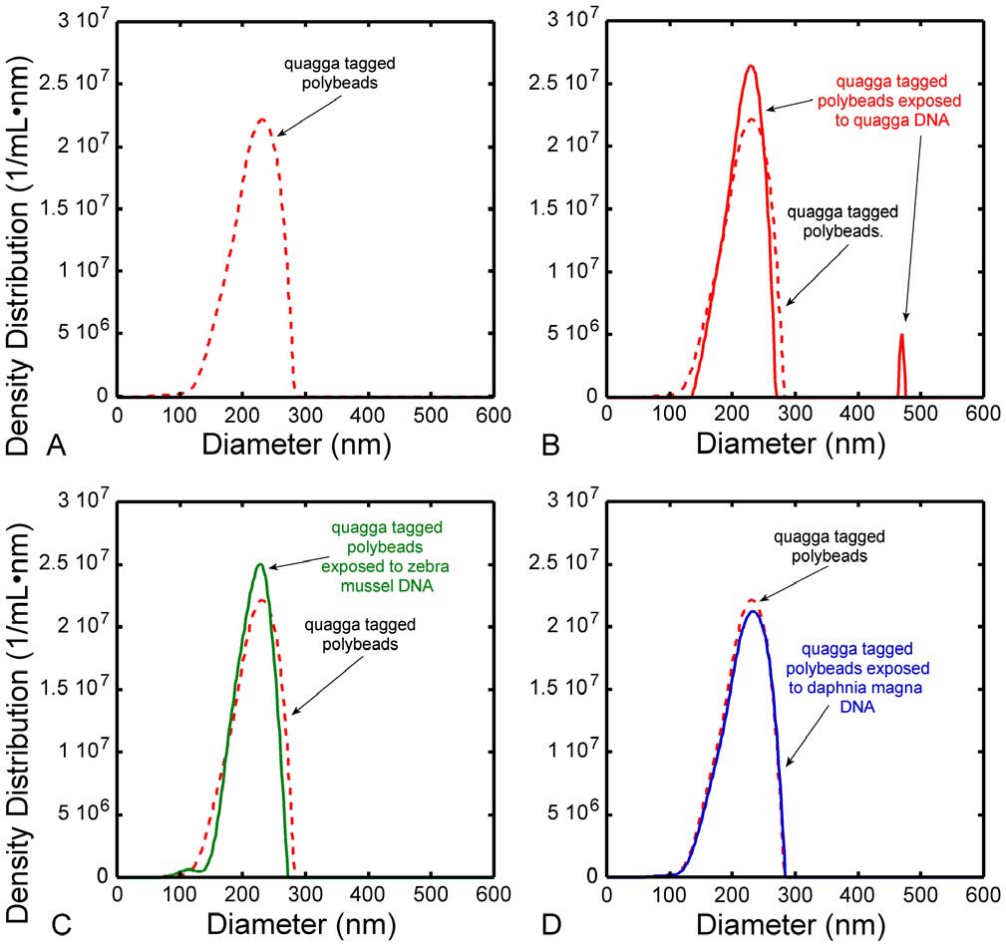
DNA detection using LTS. In A, B, C, and D the dashed red curve is the LTS particle size distribution of beads functionalized with quagga mussel tags. In B the solid red curve is the LTS particle size distribution obtained after quagga functionalized beads were exposed to denatured quagga mussel DNA where target DNA hybridization is indicated by the peak at 468 nm. In C and D the solid green and solid blue curves are the particle size distributions after quagga functionalized beads were exposed to denatured non-target DNA, zebra mussel and cladoceran respectively, where the absence of particles at larger sizes indicates a null response to non-target DNA.

First, Fig. 4A shows results for the control sample where tagged functionalized beads unexposed to DNA are seen to have a maximum in the particle-size distribution at 230 nm. As expected, note that with the tags attached, the LTS particle size distribution has shifted slightly and is broader than for the carboxylated beads alone (Fig. 2). Next, Fig. 4B shows that after exposure to target DNA some tagged beads increased in size after hybridization, producing a new peak in the particle-size distribution at 468 nm, indicating positive DNA detection of the target species. As indicated by the ratio of the areas under each peak, approximately 2 percent of the beads hybridized with the target DNA. This was likely due to an excess of functionalized beads, whereby not all functionalized beads were hybridized. The results of Rivetti and Codeluppi [26] imply that the amplified PCR product, here the mitochondrial COI fragment from quagga mussel, should remain flexible in solution [S27], which would account for the observed size of 468 nm, an increase of 238 nm. In contrast, Figs. 4C and 4D show the results for tagged beads exposed to the DNA of non-target species. In both cases, LTS gives a similar particle-size distribution with only a single peak at 230 nm, indicating negative DNA detection results for both cases.

Denatured fragments (ssDNA 600 neuclotides long) free floating in solution were also measured with LTS. The results showed that the fragments had an average diameter of ∼150 nm and that the distribution had a full width at half maximum ∼50 nm. In addition, the concentration of suspended fragments in this sample was measured with LTS to be 9×10^8^ particles/mL (∼5.4×10^11^ nucleotides/mL), orders of magnitude less that is required by established DNA detection techniques. Using simple geometrical models based on area and volume change for the beads before and after attachment of the DNA by hybridization, we can reasonably assume that the number of attached fragments ranges from 10 to 30. Because we see a narrow size distribution for particles with bound DNA, we assume that a well-defined significant fraction of each particle was coated with DNA. If the beads were not consistently hybridized, we believe there would be a correspondingly broad distribution for the second peak.

## Discussion

Our results show that laser transmission spectroscopy (LTS) can be used as a generalized method for quantitative and rapid species-specific DNA detection, and has the potential to distinguish genetic variations within a given species (e.g., different genetic populations of organisms, strains, etc.). Specifically, LTS in conjunction with functionalized nanobeads can successfully discriminate species-specific target DNA from closely related non-target DNA. Two closely related species, both invasive to North American freshwater systems (*Dressina bugensis* and *D. polymorpha*) and a common planktonic cladoceran (*Daphnia magna*) were used to demonstrate the selectivity of LTS as a DNA detection method. The technique therefore has the potential to serve generally as a means of detecting DNA from any source or distinguish genetic variation within a given species or strain of pest or pathogen. With this work, we have demonstrated the basic premise of DNA detection by LTS in the laboratory. The LTS technique has benefits over established DNA detection techniques in that it takes only a few seconds to genetically score a sample for species presence/absence, the required concentration of DNA in the sample is orders of magnitude less, and in our experience is much more cost effective than current quantitative PCR technology. Future work will clarify the broad utility of LTS, transition current lab-based success to the field, and quantify sensitivity by determining the lower concentration bounds for DNA detection by LTS. Because LTS appears to have resolving, selective, and quantitative abilities that exceed those of DLS, our future work will investigate the possibility of eliminating the need for PCR or significantly reducing the number of PCR steps required for DNA detection. The reduction or elimination of a requisite PCR step has the potential to make LTS a powerful new addition to the DNA detection arsenal.
